# Case Report: Optical coherence tomography angiography findings in radiation retinopathy

**DOI:** 10.12688/f1000research.122952.1

**Published:** 2022-08-22

**Authors:** Wafa Ammari, Asma Zaghdoudi, Olfa Berriche, Riadh Messaoud

**Affiliations:** 1 Ophthalmology, University Hospital Taher Sfar, Mahdia, 5100, Tunisia; 2 Internal Medicine, University Hospital Taher Sfar, Mahdia, 5100, Tunisia

**Keywords:** Superficial retinal capillary plexus, Deep retinal capillary plexus, Ischemic cascade, Radiation retinopathy, OCT angiography

## Abstract

We report the observation of a 31-year-old patient followed for a nasopharyngeal carcinoma since 2009, treated by locoregional radiotherapy, with a cumulative dose of 70 Gray.  This case presented all ocular complications of radiotherapy; radiation retinopathy (RR), which is the most severe complication, could be diagnosed earlier and have a better prognosis if optic coherence tomography angiography (OCTA) was performed.

She presented with a progressive decline in bilateral visual acuity. Ophthalmologic examination revealed bilateral posterior subcapsular cataract, radiation retinopathy, and optic neuropathy. The OCT B-scan showed more pronounced macular edema in the right eye.  The OCTA revealed enlargement of the central avascular zone and loss of the deep and superficial retinal vascular network. The patient received three consecutive monthly intravitreal injections of anti-vascular endothelial growth factor (VEGF), without improvement in visual acuity.

The aim of this case report is to present the contribution of OCT-A in the diagnosis of radiation maculopathy, and attribute these changes to ischemia at the level of the retinal vascular network.

## Introduction

Locoregional radiotherapy is the most effective treatment against nasopharyngeal carcinoma (NPC). ^1^ The proximity with orbit tissues exposes them to severe damages. ^2^ However, late-onset, sight-threatening ocular complications may occur, including cataract, optic neuropathy, radiation retinopathy (RR), and ocular surface disease. ^2^ The early diagnosis of these lesions allowed to better prognosis. ^1^ The optical coherence tomography angiography (OCT-A) allowed to investigate neovascular alteration for patients suffering from RR even before the inset of loss of vision. ^1^

We report a case of radiation retinopathy in a 31-year-old female with NPC, treated by locoregional radiation therapy (LRT). She presented all post-radiotherapy ocular complications with late diagnosis of RR and poor prognosis.

The purpose of this case report is to analyze the findings and the usefulness of OCT-A.

## Case report

**Figure 1.**
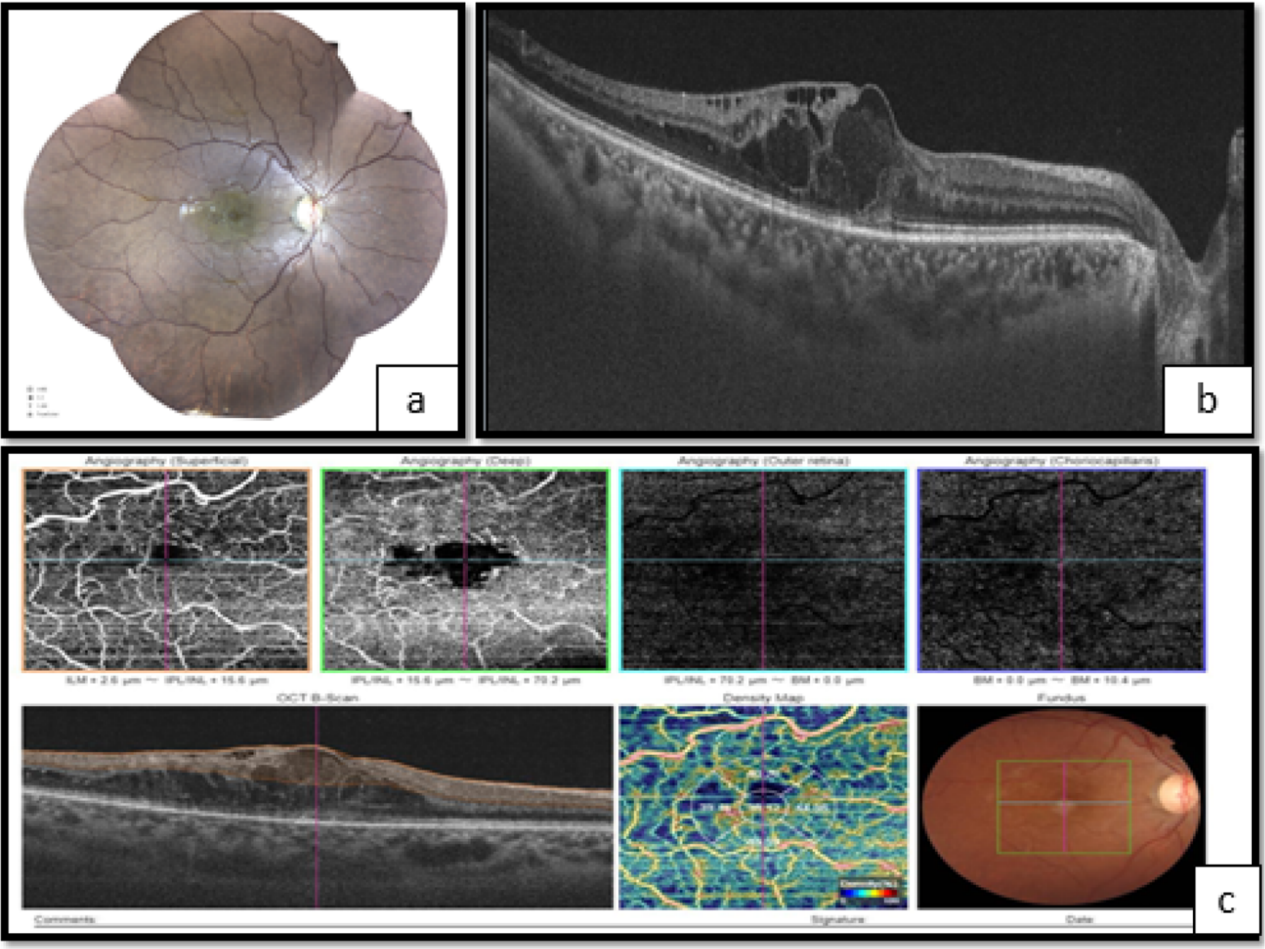
Right eye. a. Fundoscopy showing vascular tortuosity and dilation of peripheral retinal vessels, the disappearance of foveolar reflection, and mild pallor of the optic disc. b. Optical coherence tomography (OCT) showed macular edema. c. Optical coherence tomography angiography (OCT-A) showing enlargement of the centralavascular zone, and hypoperfusion of both superficial and deep retinal capillary network.

**Figure 2.**
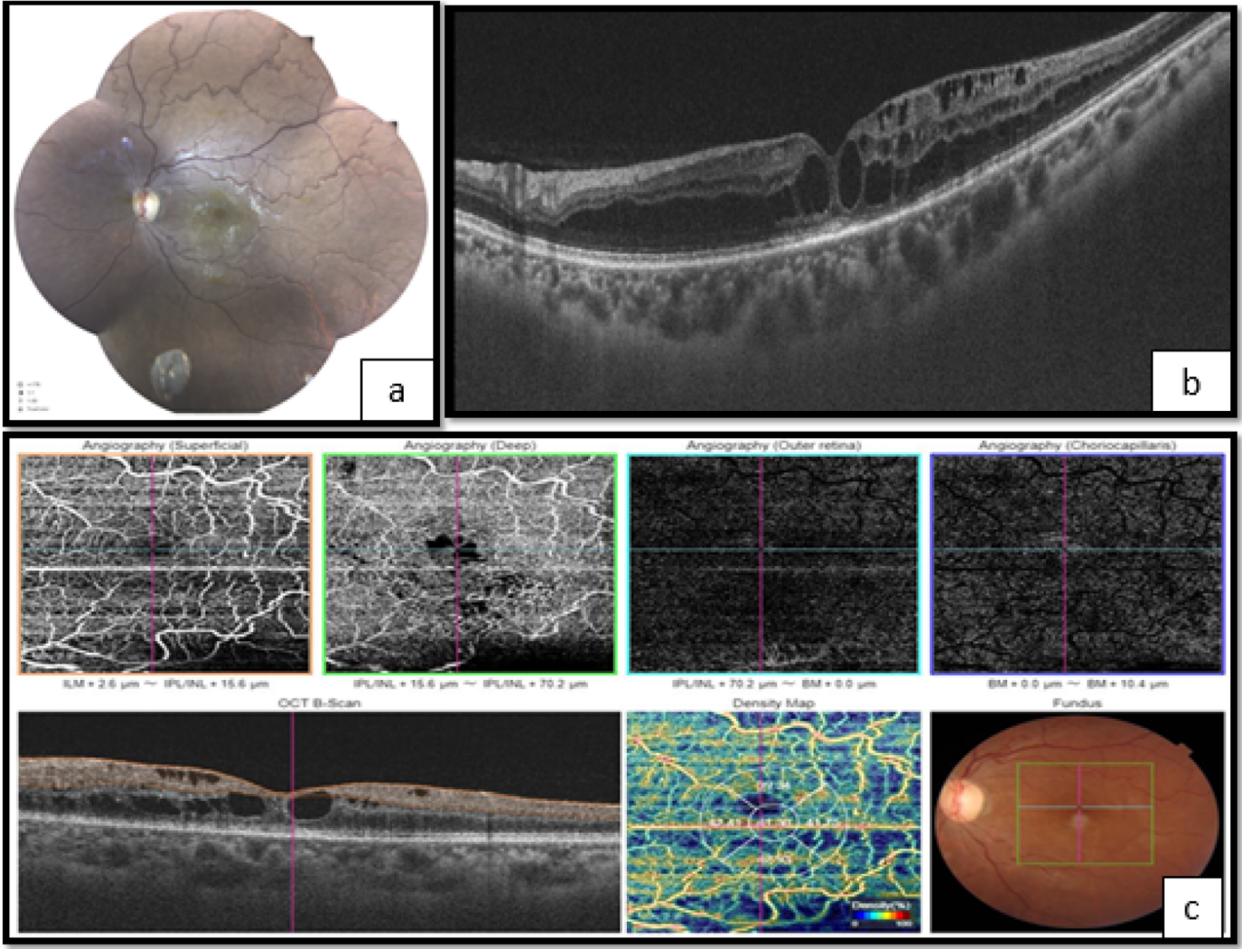
Left eye. a. Fundoscopy showing vascular tortuosity and dilation of peripheral retinal vessels, the disappearance of foveolar reflection, and mild pallor of the optic disc. b. The optical coherence tomography (OCT) shows macular edema. c. Optical coherence tomography angiography (OCT-A) showing enlargement of the central avascular zone, and hypoperfusion of both superficial and deep retinal capillary network.

A 31-year-old Tunisian, unemployed female with a history of nasopharyngeal carcinoma was diagnosed in 2009 and treated by locoregional radiotherapy. The overall administered dose was about 75 Gy. She presented with adrenal insufficiency, hypothyroidism, and osteonecrosis as side effects of the treatment. She complained of progressive painless loss of vision in both eyes. On examination, her best-corrected visual acuity was 20/40 in both eyes. The ocular motility was full, and no afferent pupillary defect was noted. A symmetrical subcapsular cataract was found. The rest of the anterior segment examination was unremarkable. No vitreous cells were noted. Fundoscopy showed microvascular changes mainly marked by vascular tortuosity and microaneurysms, optic disc pallor, and decreased foveal reflex. Fluorescein angiography was not performed because the patient was allergic to fluorescein. OCT in B-scan showed bilateral macular edema with a central macular thickness of, respectively, 532 μm in the right eye (RE), and 406 μm in the left eye (LE). OCT angiography (OCT-A) disclosed enlargement of the central avascular zone, and hypoperfusion of both superficial and deep retinal capillary networks ( Figures 1 and 2). The vessel density was reduced to 38.12 % in the inferior macular area of the RE, and to 39.34 % in the superior macular area of the LE. A systemic workup was performed to rule out other causes of ischemic retinopathy (diabetes mellitus, blood dyscrasias, and carotid insufficiency). Based on medical history, ocular findings, and negative systemic workup the diagnosis of radiation retinopathy was finally established. After informed consent, and a negative pregnancy test, the patient underwent three monthly intravitreal bevacizumab injections (1.25 mg). The improvement of visual acuity was poor.

## Discussion

Radiation retinopathy (RR) was first described in 1933 by Stallard, ^3^ as a predictable complication of radiation exposure. It most commonly occurs between six months and three years after irradiation. ^4^ In this case, the diagnosis was later, twelve years after irradiation. A higher total radiation dose is the highest risk factor, as the incidence of RR increases at doses greater than 45 Gy. This patient received 75 Gy. Histopathological studies have illustrated a vasculopathy with the destruction of the endothelial cells followed by vascular occlusion and capillary dropout. ^3^^,^^4^ The microvascular alterations are associated with a reduction of retinal oxygenation, blood flow, and ischemia. ^2^^–^^4^ Contrast sensitivity decrease and visual field impairment were notified in patients treated with radiotherapy. ^1^ Our patient had gradually decreased bilateral visual acuity, as well as cataract and optic neuropathy. The clinical appearance mimics many lesions of diabetic retinopathy such as microaneurysms, macular edema, cotton-wool spots, retinal neovascularization, vitreous hemorrhage, and tractional retinal detachment. ^4^ The main tests usually performed on patients are fluorescein fundus angiography (FFA) and optical coherence tomography (OCT). The first exam hallmarks are capillary dilatation and microaneurysms, frequently in combination with ischemia or macular edema. ^5^ On OCT images, we found a disappearance of the macular depression with macular edema, a significant thinning of the inner plexiform, inner nuclear, and outer plexiform layers. ^5^ However, FFA is an invasive diagnostic technique. Intravenous dye injection used may cause severe anaphylaxis, particularly in immunocompromised patients. It was not performed on our patient. Besides, OCT cannot capture vessel network status. Recently, OCT-A, has shown to be a safe and non-invasive examination that combines traditional OCT and FFA. It can provide high-resolution images of each layer of the retina and quantify the retinal microvascular networks without the use of exogenous dyes. OCTA has been introduced for the detection of subtle microvascular changes in radiation retinopathy. ^6^^,^^7^ Vascular abnormalities are manifested by an enlargement of the central avascular zone and a reduction of vessel density in the deep vascular plexus of the foveal area. Whereas it’s less reduced in superficial layers. The susceptibility of the deep layer can be explained by the direct connection of the superficial capillary plexus to the retinal arterioles with greater perfusion and oxygen supply. ^3^ This change in structure can be explained by direct compression of the retinal vascular network, deep in the first place, by intra-retinal fluid cysts. Li *et al.* ^1^ found that OCTA detects early vascular alterations of the retina in patients with normal-ranged visual acuity. It provided a quantitative measurement of retinal capillary changes which may predict future development of radiation-induced retinal toxicity. ^5^ They suggested the implementation of OCTA for the early detection and consistent monitoring of RR. In this sense, a grading system was proposed based on clinical findings in OCTA, increased central macular thickness, evident cysts, and ophthalmoscopy findings. ^5^ The disadvantage is the presence of several artifacts, especially after treatment.

Furthermore, due to the clinical and pathophysiological similarities with diabetic retinopathy, it inspired the treatment of radiation retinopathy. ^8^ Initially, treatments were based on the use of retinal laser. ^8^ Sector photocoagulation improves clinical signs, but the visual outcome is poor. ^8^ Intravitreal injection of anti-VEGF or corticosteroids has been shown to improve visual acuity, reduce cystoid macular edema, and the risk of the development of radiation retinopathy. ^3^^,^^8^ The visual acuity of our patient didn’t change, probably because she presented with several complications of local radiotherapy, such as cataract and optic neuropathy, and ischemia affecting deep layers. Continuous treatment is necessary to maintain acuity improvement ^7^; this requires good patient adherence. ^8^ The optimal regimen for anti-VEGF therapy is not yet identified. ^7^ There have been recent preventive efforts to avoid signs that radiation damage has already occurred, particularly since there is still no curative treatment. ^9^

## Conclusions

Radiation retinopathy manifests itself on OCTA by an enlargement of the foveolar avascular zone and a rarefaction of the vascular network at the level of the deep and vascular networks, even in eyes without clinical evidence of radiation retinopathy.

## Data availability

All data are included as part of the article and no additional data are required.

## Consent

The patient has consented to the submission of the case report for submission to the journal.
