# Supplementary material for: Case Report: Optical coherence tomography angiography findings in radiation retinopathy
Source: F1000Res. 2023 Sep 25;11:968. Originally published 2022 Aug 22. [Version 3] doi: 10.12688/f1000research.122952.3 (PMC10523097; doi:10.12688/f1000research.122952.3)
Supplement: Peer review history

# Reviewer report: Reviewer response for version 3

**DOI:** 10.5256/f1000research.156359.r209186

**Published:** 2023-09-26

**Authors:** David Solá-Del Valle

**Affiliations:** 1 Department of Ophthalmology, Glaucoma Service, Massachusetts Eye and Ear Infirmary, Boston, MA, USA

They improved the report to my satisfaction.

Are enough details provided of any physical examination and diagnostic tests, treatment given and outcomes?

Yes

Is the case presented with sufficient detail to be useful for other practitioners?

Yes

Is sufficient discussion included of the importance of the findings and their relevance to future understanding of disease processes, diagnosis or treatment?

Yes

Is the background of the case’s history and progression described in sufficient detail?

Yes

Reviewer Expertise:

Ophthalmology and glaucoma

I confirm that I have read this submission and believe that I have an appropriate level of expertise to confirm that it is of an acceptable scientific standard.

# Reviewer report: Reviewer response for version 3

**DOI:** 10.5256/f1000research.156359.r209187

**Published:** 2023-09-26

**Authors:** Helmi Ben Saad

**Affiliations:** 1 Faculty of Medicine of Sousse, Laboratory of Physiology, University of Sousse, Sousse, Tunisia

The authors have improved the quality of their paper. The paper can be published in its actual form.

Are enough details provided of any physical examination and diagnostic tests, treatment given and outcomes?

Yes

Is the case presented with sufficient detail to be useful for other practitioners?

Yes

Is sufficient discussion included of the importance of the findings and their relevance to future understanding of disease processes, diagnosis or treatment?

Yes

Is the background of the case’s history and progression described in sufficient detail?

Yes

Reviewer Expertise:

Medical writing skills; physiology

I confirm that I have read this submission and believe that I have an appropriate level of expertise to confirm that it is of an acceptable scientific standard.

# Reviewer report: Reviewer response for version 2

**DOI:** 10.5256/f1000research.142080.r188589

**Published:** 2023-07-26

**Authors:** Helmi Ben Saad

**Affiliations:** 1 Faculty of Medicine of Sousse, Laboratory of Physiology, University of Sousse, Sousse, Tunisia

I read the revised version of the case report entitled “Case Report: Optical coherence tomography angiography findings in radiation retinopathy”.

 First, again the case report is interesting. However, I am really surprised by the fact that the authors have not to corrected their paper according to my previous remarks, which were sent almost 1 year ago (50% of my previous remarks were neglected). I have no explication to this situation.

 If an author refuses to apply the recommendations made by a reviewer, the author should provide a detailed and well-reasoned response explaining their rationale. The response should address each point raised by the reviewer and explain why the author believes their current approach is valid. It is essential for the author to be respectful and professional in their response, as well as to back their arguments with relevant evidence or references when applicable. In some cases, there may be legitimate reasons for an author to disagree with certain recommendations, such as limitations of the study, available resources, or differences in interpretation. However, it is important to note that the review process aims to improve the quality of the paper, and authors should carefully consider reviewer feedback before making their final decision.

 Below if my review, (almost the one I sent 1 year ago).

**A.** I recommend that authors apply the CARE (Consensus-based Clinical Case Reporting) guidelines ( **see reference 1 below**).

 The CARE guidelines are designed to improve the quality and transparency of clinical case reports. The benefits of using the CARE guidelines, are outlined in the article. 

 Overall, the CARE guidelines offer a comprehensive and standardized approach to reporting clinical case reports, which can lead to improved transparency, quality, and educational value, benefiting both researchers and healthcare professionals.

**B. **The authors are also asked to full in (and submit) the CARE checklist (see reference 2 below)

**C. ABSTRACT**

 I recommend that authors apply the IOC: Introduction, observation, Conclusion

 L5: we found radiation retinopathy (RR), and l11 we should find RR not radiation retinopathy

 Sometimes OCTA (line 8) and line 18, we note OCT-A (is it OCTA or OCT-A?)? Use one expression and avoid elegant variation of terms

 L9: Change She presented by The patient presented

 L16: delete (VEGF) used one time in the abstract

**D.** All abbreviations should be explained the first time they are used - unless it is a standard unit of measurement - and thereafter the use of abbreviations should be consistent throughout the paper. Avoid excessive use of abbreviations.

 For examples,

**E. **The paper should be proofread (this is a MAJOR remark).

**F**. **MANUSCRIPT**

 *In the beginning of the section Case Report, the authors are asked to write the following sentence:

 This case report followed the CARE guideline (add the reference)

 *The patient received 70 Gy (see the abstract) or 75 Gy (see the observation and the discussion)? More rigor is needed.

 *Change the sentence “fluorescein fundus angiography (FFA) and optical coherence tomography (OCT).” by “fluorescein fundus angiography (FFA) and OCT.”

 *Inside the text we find Zihing 1 (but in reference 1, we note Zijing): so is it Zihing or Zijing.

 *VEGF was not defined in the text (the abstract and the text are 2 different documents)

 Please add a subsection called: Limitations

**References**

1. Gagnier JJ, et al The CARE Guidelines: Consensus-based Clinical Case Reporting Guideline Development. Glob Adv Health Med. 2013 Sep;2(5):38-43. doi: 10.7453/gahmj.2013.008. PMID: 24416692. https://journals.sagepub.com/doi/pdf/10.7453/gahmj.2013.008
2. CARE Checklist of information to include when writing a case report: https://static1.squarespace.com/static/5db7b349364ff063a6c58ab8/t/5db7bf175f869e5812fd4293/1572323098501/CARE-checklist-English-2013.pdf

Are enough details provided of any physical examination and diagnostic tests, treatment given and outcomes?

Yes

Is the case presented with sufficient detail to be useful for other practitioners?

Yes

Is sufficient discussion included of the importance of the findings and their relevance to future understanding of disease processes, diagnosis or treatment?

Yes

Is the background of the case’s history and progression described in sufficient detail?

Yes

Reviewer Expertise:

Medical writing skills; physiology

I confirm that I have read this submission and believe that I have an appropriate level of expertise to confirm that it is of an acceptable scientific standard, however I have significant reservations, as outlined above.

## References

[1] : The CARE Guidelines: Consensus-based Clinical Case Reporting Guideline Development. *Glob Adv Health Med* .2013;2(5) : 10.7453/gahmj.2013.008 38-43 10.7453/gahmj.2013.008 24416692PMC3833570

[2] : Checklist of information to include when writing a case report. Reference source

# Reviewer report: Reviewer response for version 1

**DOI:** 10.5256/f1000research.135008.r165708

**Published:** 2023-04-11

**Authors:** David Solá-Del Valle

**Affiliations:** 1 Department of Ophthalmology, Glaucoma Service, Massachusetts Eye and Ear Infirmary, Boston, MA, USA

Overall, it's a well written report. One small discrepancy between the dose of Gray received (70 or 75?) in abstract and body of paper. I wish the introduction had a paragraph on OCTA (how does it work? and how is it different from regular OCT) for the general practitioner. I would encourage the writers to proof read the report one more time for typographical errors and syntax (ensure reading flows well, especially the abstract). It would also be important to better summarize data on OCTA in the discussion, especially data from trials or large studies that could be relevant to this patient.

Are enough details provided of any physical examination and diagnostic tests, treatment given and outcomes?

Yes

Is the case presented with sufficient detail to be useful for other practitioners?

Yes

Is sufficient discussion included of the importance of the findings and their relevance to future understanding of disease processes, diagnosis or treatment?

Yes

Is the background of the case’s history and progression described in sufficient detail?

Yes

Reviewer Expertise:

Glaucoma/Ophthalmology

I confirm that I have read this submission and believe that I have an appropriate level of expertise to confirm that it is of an acceptable scientific standard, however I have significant reservations, as outlined above.

# Response

**Published:** 2023-05-21

**Authors:** WAFA AMMARI

**Affiliations:** University of Medecine Monastir, Tunisia

Thank you for your interest to this article and for your precious remarks.

 The total dose was 75 Gray. We had introduced a paragraph about the difference between regular OCT and OCTA in introduction.  We had reported the necessary correction for the typographical errors and syntax and we had mad changes for abstract. There are not yet large studies in this topic (The OCT angiography findings in radiation retinopathy).

# Reviewer report: Reviewer response for version 1

**DOI:** 10.5256/f1000research.135008.r148253

**Published:** 2022-08-31

**Authors:** Helmi Ben Saad

**Affiliations:** 1 Faculty of Medicine of Sousse, Laboratory of Physiology, University of Sousse, Sousse, Tunisia

I read with great interest the case-report entitled “Case Report: Optical coherence tomography angiography findings in radiation retinopathy”. The rationale of the study is interesting. However, a revision is needed.

 Here are my remarks:

**GENERAL REMARKS**

1. I recommend that authors apply the CARE guidelines ^1^.
2. For the abstract, I recommend that authors apply the IOC plan in the abstract: Introduction, observation, conclusion.
3. All abbreviations should be explained the first time they are used - unless it is a standard unit of measurement - and thereafter the use of abbreviations should be consistent throughout the paper. Avoid excessive use of abbreviations.
4. The paper should be checked by a person fluent in English.
5. Sometimes Gy sometime Gray: please use one term
6. The patient received 70 Gy (see the abstract) or 75 Gy (see the observation and the discussion)?

**SPECIFIC REMARKS**

**1. Abstract:**

 Some corrections are needed. For example write ‘A 31 year-old female”, delete (RR) and (VEGF), write “The patient presented with…”. Please use OCTA or OCT-A not both.

**2. Manuscript:**

- Avoid abbreviating LE and RE, instead using full text: left-eye, right-eye.
- Misuse of abbreviations: for examples, write “*We report a case of RR in a 31-year-old”, “history of NPC was”, “treated by LRT”, OCT-A disclosed enlargement’, “diagnosis of RR was finally”, “RR was first described in 1933”, “angiography (FFA) and OCT”, “microvascular changes in RR”, “the treatment of RR8”, “of RR. 3,8 The visual”, “complications of LRT”, “RR manifests itself”, “clinical evidence of RR”.
- More precision is needed: for example, L3 of the Case report, write ‘The patient complained from “ rather than “She complained from progressive’. In this sentence (L11page 5/6) “retinopathy, it inspired”: it refers to what?
- In the beginning of the case report, please mention the following sentence “This observation was reported according to the CARE guidelines (ref)”.
- Some mistakes related to references: for examples you wrote “by Stallard,3”, but reference 3 is the one of Rose K *et al.* You also wrote “Li *et al* ^1^ found”, however the reference number 1 is of Zijing *et al*?
- Some sentences are lacking references: for example, add a reference for these sentences: “A higher total radiation dose is the highest risk factor, as the incidence of RR increases at doses greater than 45 Gy”, “However, FFA is an invasive diagnostic technique.”, “Intravenous dye injection used may cause severe anaphylaxis, particularly in immunocompromised patients”.

**3. References:**

 Please verify all your references, especially references number 1 and 3.

**4. Figures:**

 Figure 1 Title: c. OCT angiography showing….

 Figure 2 Title: c. OCT angiography showing….

Are enough details provided of any physical examination and diagnostic tests, treatment given and outcomes?

Yes

Is the case presented with sufficient detail to be useful for other practitioners?

Yes

Is sufficient discussion included of the importance of the findings and their relevance to future understanding of disease processes, diagnosis or treatment?

Yes

Is the background of the case’s history and progression described in sufficient detail?

Yes

Reviewer Expertise:

Medical writing skills; physiology

I confirm that I have read this submission and believe that I have an appropriate level of expertise to confirm that it is of an acceptable scientific standard, however I have significant reservations, as outlined above.

## References

[1] : The CARE Guidelines: Consensus-based Clinical Case Reporting Guideline Development. *Glob Adv Health Med* .2013;2(5) : 10.7453/gahmj.2013.008 38-43 10.7453/gahmj.2013.008 24416692PMC3833570

# Response

**Published:** 2022-09-02

**Authors:** WAFA AMMARI

**Affiliations:** University of Medecine Monastir, Tunisia

**Thank you for the remarks and the attention paid to this original article.**

**General remarks**

 1 and 2 we followed the journal recommendations.

 3. All abbreviations were explained first time they are used. In ophthalmology, we always used abbreviations.

 4. The paper was checked by newspaper experts who appreciated the English used.

 5.Gy and Gray are both used, but we rectified this.

 6.The patient received 75 Gy. It was a keyboard typo for the abstract.

**Specific remarks:**

**1.Abstract:**

**2.Manuscript:**

 Stallard is the first person to describe this entities and this is mention in the article of Rose K et al.

 The Author’s name is Zijing Li

**3.References:**

 All references were verified

**4. Figures:**

 The figures showing multimodal imaging. The OCT-A is the c only.

**For the other remarks, they have been  corrected on the article.**
